# Revised recommendations on standards and norms for palliative care in Europe from the European Association for Palliative Care (EAPC): A Delphi study

**DOI:** 10.1177/02692163221074547

**Published:** 2022-02-03

**Authors:** Sheila Payne, Andrew Harding, Tom Williams, Julie Ling, Christoph Ostgathe

**Affiliations:** 1 International Observatory on End of Life Care, Faculty of Health and Medicine, Lancaster University, Lancaster, UK; 2 European Association for Palliative Care, Vilvoorde, Belgium; 3 Friedrich-Alexander-Universität Erlangen-Nürnberg (FAU), Department of Palliative Medicine, Comprehensive Cancer Center Erlangen-EMN (CCC ER-EMN), Universitätsklinikum Erlangen, Erlangen, Germany

**Keywords:** Palliative care, end-of-life care, Delphi technique, surveys and questionnaires, Delivery of Health Care

## Abstract

**Background:**

In 2009, the EAPC published recommendations on standards and norms for palliative care in Europe, and a decade later, wished to update them to reflect contemporary practice.

**Aim:**

To elicit consensus on standards and norms for palliative care in Europe, taking account of developments since 2009.

**Design:**

A Delphi technique used three sequential online survey rounds, and a final expert consultation (EAPC Board). The original 2009 questionnaire with 134 statements was updated with 13 new concepts and practices following a scoping of the literature between 2009 and 2020 (total: 147 statements).

**Setting/participants:**

One contact of Boards of 52 national European organisations affiliated to the EAPC were invited to participate, with subsequent rounds sent to respondees. The EAPC Board (*n* = 13) approved final recommendations.

**Results:**

In Round 1: 30 organisations (14 organisations × two people, 16 organisations × one person, total *n* = 44) in 27 countries responded (response rate 58% organisations, 82% countries**)**, Round 2 (*n* = 40), Round 3 (*n* = 38). 119 statements reached consensus in Round 1, 9 in Round 2, 7 in Round 3. In total 135/145 statements in five domains (terminology, philosophy, levels, delivery, services) reached consensus (defined as >75% agreement), (122) were original EAPC recommendations with 13 new recommendations included emerging specialisms: neonatal, geriatric and dementia care, and better care practices. Seven statements failed to reach consensus and four were removed as irrelevant or repetition.

**Conclusions:**

Most recommendations on standards and norms for palliative care in Europe remain unchanged since 2009. Evolving concepts in palliative care can be used to support advocacy.

**What is already known about the topic?**

- The EAPC published recommendations on standards and norms in palliative care for Europe in 2009.
- The Delphi technique is a well recognised way to elicit the views of stakeholders and obtain consensus.
- There are a diversity of international and national definitions and concepts in palliative care which makes comparison between countries and delivery of health care complex.

**What this paper adds?**

- The majority (122) of standards and norms in five domains (definitions of palliative care, philosophy, levels, delivery, services) in palliative care in Europe have remained unchanged over the last decade.
- 13 new standards and norms reached consensus, relating to emerging specialisms such as neonatal, geriatric and dementia palliative care, and recommendations for better access to national information sources and the use of digital health records.

**Implications for practice and research?**

- New recommendations recognise that there are emerging subspecialisations in palliative care in the fields of neonatal paediatrics and geriatric medicine indicating that care extends across the lifespan.
- New recommendations also have implications for service quality improvements including enhancing open visiting, availability of essential medicines, better information exchange, including digital medical records and access to specialist equipment.
- Future research and clinical care needs to include multiple domains to assess quality improvements in palliative care.

## Background

Concepts and definitions in palliative care have evolved over time and differ across geographical regions.^1–3^ This diversity may lead to confusion for policy makers, service providers and service users.

The World Health Organization (WHO) initially defined palliative care in 1990, and subsequently updated this in 2002.^4^ This definition remains unchanged two decades later despite palliative care services developing significant diversity, and WHO’s endorsement of models of integration of palliative care into national person-centred healthcare systems.^5,6^ There have been chronological developments in establishing definitions from national, regional and global perspectives and national standards published by a few countries such as England and Wales, USA, Australia and New Zealand.^7^ In 2009, the EAPC published recommendations on standards and norms for the delivery of palliative care services in Europe.^8,9^ Their rationale for cross-country standards and norms included promoting uniformity, enhancing collaboration, facilitating quality improvement and fostering performance measurement.^8,9^ They aimed to generate common terminology and standards across Europe, thus providing guidance to service providers, stakeholders and decision makers. More recently some organisations have made global recommendations on definitions, but arguably considerable diversity remains. The Lancet Commission proposed the adoption of the concept of addressing ‘severe health-related suffering’ to encapsulate the objective of palliative care on a global basis.^10^ However, their emphasis on physical and psychological suffering arguably marginalises social and spiritual domains of palliative care provision. More recently, the International Association for Hospice and Palliative Care (IAHPC) published a new consensus based definition of palliative care drawing also upon the notion of ‘severe health-related suffering’ which has not been widely endorsed or accepted.^11^

The original EAPC recommendations have been influential in the development of palliative care and widely cited,^8,9^ but are over a decade old. Evidence of rapid developments and new practices in Europe^12^ indicate that they should be updated. The aim was to update the previously published standards and norms for palliative care in Europe, taking account of recent evidence about new practices and developments.

## Methods

The main aim was to elicit consensus on standards and norms for palliative care in Europe, taking account of developments since 2009.^8,9^ An international team comprised of experts in palliative care from three countries (Belgium, Germany and the United Kingdom) used a three-round Delphi technique to establish consensus on revisions to standards and norms in palliative care in Europe. We built upon the original EAPC standards and norms recommendations^8,9^ by searching the literature to identify new developments and areas of practice between 2009 and 2020. A structured three-round Delphi technique^13^ was used to elicit consensus on original and new standards and norms, followed by a final expert consultation where the multidisciplinary EAPC Board of Directors were invited to provide qualitative comments on the outcomes of the structured rounds (Directors listed in the Acknowledgements). The Delphi technique is a formal consensus procedure which allows participants to rate their level of agreement to a statement, thus shaping a consensus.^13^ The structured rounds offered anonymity to respondents (preventing potential influences of group conformity), iteration (allowing respondents to change their opinions between rounds) and individualised feedback (communicating the mean results of the previous round and individual responses). The study adhered to the CREDES recommendations on methodology and reporting of Delphi studies,^14^ and online survey methodology observed the Checklist of Reporting Results of Internet E-Surveys.^15^

### Scoping of the literature

A scoping of the literature is a versatile and flexible approach that seeks to map key areas of research and concepts, using a broad search strategy, followed by charting and summarising of the data.^16^ A literature search was conducted during March 2020 to locate peer reviewed publications using the search terms ‘Palliative care; palliative medicine; end of life care; hospice care; terminal care’. We searched Turning Research into Practice (TRIP) database, as recommended by a specialist librarian, for these key words in the title or anywhere in the text. Inclusion criteria were: published 1st January 2009 to 1st March 2020, systematic reviews, empirical research, controlled trials, English language, publically available. Exclusion criteria were: opinion pieces, unpublished, not in English. Searches resulted in 1002 ‘hits’. After duplicate removal, 850 sources were screened by title and abstract (AH, TW), with 16 full texts discussed (SP, AH, TW). The original EAPC standards and norms^8,9^ were mapped onto a matrix, and upon reading each paper we identified whether the paper contained sufficient material to amend an existing standard and norm, or whether it had sufficient scope to add a new one under the respective five domains (see Supplemental Information 1). Data were extracted from the selected publications and used to ‘chart’ against the matrix. Where new concepts or practices, relevant to the European context, were identified, these were summarised as new statements for inclusion in the Delphi procedure.

### Population, sample and recruitment

As the EAPC commissioned an update of the previous recommendations, rather than a completely new study, we used the same population, sampling and recruitment procedures to ensure that the findings were comparable.^8,9^

*Population*: Boards members of 52 national and professional organisations affiliated to the EAPC from 33 countries across Europe, and 13 Board Members of the EAPC.

*Sample*: Rounds 1–3: One key contact of organisations were identified by the EAPC. Expert consultation: 13 members of EAPC Board.

*Recruitment*: To comply with General Data Protection Regulation legislation, initial emailed invitations to Round 1 were distributed by the EAPC Head Office (November 2020), with all responses and rounds 2 (January 2021), 3 (March 2021) managed by AH. As an incentive for completing the study, participants who completed all rounds were entered into a draw for a free registration for the EAPC World Congress in 2021. JL distributed the final consultation document to all EAPC Board members (May 2021) and provided collated responses to AH.

### Original questionnaire revisions

The original 2009 questionnaire with 134 statements was updated with 13 new concepts and practices identified following scoping of the literature between 2009 and 2020 (147 statements in total). The structure of the original questionnaire was used with: 145 statements in five sections (terminology (7 original), philosophy (9 original), levels of care (16 original, 6 new), delivery (56 original, 2 new) and services (44 original, 5 new)) were prepared with two final open questions asking about understanding of ‘norms’ (total 147) and an opportunity to identify omissions, and in Round 1 only, a demographic section. Respondents were invited to rate their level of agreement with each statement on a five-point Likert scale (strongly disagree, moderately disagree, neither agree nor disagree, moderately agree, strongly agree, a ‘don’t know’ option was also available). In addition, 17 roles associated with palliative care were listed with a 5 point Likert scale labelled ‘very essential to inessential’. These scales were used in the previous questionnaire, thus allowing for direct comparison and consistency. Below each structured scale, there was space for open-ended comments such as revisions to wording or explanations of the scores given. Open-ended comments were all read carefully and discussed by AH, TW, SP, statements were revised or reworded for clarity. Consensus was set at a mean of 75% per statement to acknowledge the diversity of practice and contexts in Europe. Each round was kept open for 4–5 weeks, with up to two reminders sent to non-responders.

### Delphi procedure

Consensus was defined as >75% agreement. *Round 1*: In November 2020, panel members were sent an online questionnaire with 147 statements using Qualtrics software. *Round 2*: In January 2021, respondents to Round 1 were sent an online questionnaire comprising 25 statements that had failed to reach consensus in Round 1. They were provided with details of the mean score for each statement, their own previous score and any comments they had made. *Round 3*: In March 2021, respondents to Round 2 were sent an online questionnaire comprising 14 statements that had failed to reach consensus in Round 2. The survey portal remained open for 4–5 weeks, with up to two reminders sent to non-respondents. *Expert consultation*: In May 2021, the updated recommendations were sent to all EAPC Board members.

### Analysis

Descriptive statistics after each round, included statement scores, means, medians and distribution. For each statement, qualitative comments were entered into a spreadsheet; these were read and discussed by AH, SP, TW to identify semantic, practical and conceptual issues. Qualitative analysis of comments guided rewording or removal of statements. The expert consultation resulted in largely editorial comments on the manuscript.

Approval to conduct the Delphi study was obtained from the Faculty of Health and Medicine Research Ethics Committee, Lancaster University on 30th June 2020 (FHMREC19066).

## Results

### Participation in the survey (Rounds 1–3)

**Figure 1.**
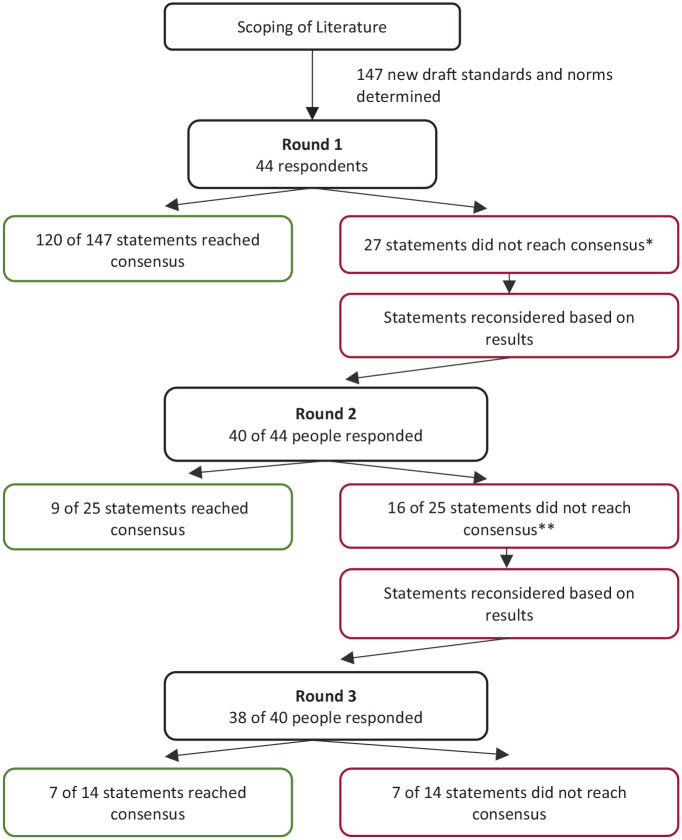
Flow chart of three round Delphi process. *Two items omitted after R1 Supportive care should not be used as a synonym of palliative care. Population served, Adequate provision of palliative care for noncancer patients requires additional resources. If non-cancer patients were to have equal access to palliative care compared to cancer patients, the percentages of patients requiring palliative care are estimated at 40% (non-cancer patients) and 60% (cancer patients) respectively. **Two items omitted after R2 Non-specialist Palliative Care, Hospital at home.

The flowchart in Figure 1 presents an overview of the Delphi technique, the number of completed responses and outcomes in each of the three structured rounds.

In Round 1, 30 organisations (14 organisations two individuals, 16 organisations one person, total *n* = 44) from 27 European countries responded (response rate: 58% organisations, 82% countries). It should be noted that in some countries, there is more than one organisation (range 1–4, median 1). In each subsequent round only those previously responding were eligible, therefore in Round 2, 40 of 44 individuals (response rate 90%) and in Round 3, 38 of 40 individuals responded (response rate 95%). The characteristics of the respondents in Round 1 are presented in Table 1.

**Table 1.** Characteristics of Delphi Round 1 respondents (*n* = 44).

Variable	*N* and percentage
Age	
Mean (SD)	52.8 (9)
Age range	34–70
Gender, *n* (%)	
Female	30 (68.2%)
Country of organisation grouped by geographical region in Europe, (organisation; number of respondents)	Western Europe Austria (1; 2) Belgium (1; 2) France (1; 2) Germany (1; 1) The Netherlands (1; 2) Republic of Ireland (1; 2) Switzerland (1; 1) United Kingdom (2; 4) Southern Europe Greece (1; 2) Israel (1; 1) Italy (1; 1) Portugal (1; 2) Spain (2; 3) Northern Europe Denmark (1; 2) Finland (1; 1) Iceland (2; 2) Norway (2; 2) Sweden (1; 1) Central and Eastern Europe Croatia (1; 1) Hungary (1; 1) Latvia (1; 2) Lithuania (1; 1) North Macedonia (1; 1) Poland (1; 1) Romania (1; 1) Slovakia (1; 1) Slovenia (1; 2)
Profession, *n* (%)	Physician 27 (55%) Nurse 7 (14.3%) Social worker 4 (8.2%) Administrator 3 (6.1%) Volunteer 1 (2%) Social scientist 1 (2%) Psychologist 1 (2%) Other 5 (10%)
Main focus of competence	Clinical 44% Research 26% Policy/Administration 26% Other 4%
Total professional experience in years	
Mean (SD)	24.9 (8.2)
Range	6–42

### Consensus on statements

In total 135/145 statements reached consensus (defined as over 75% agreement); with 118 statements reaching consensus in Round 1, nine in Round 2, and seven in Round 3 (see Tables 2–4). Of these, the majority (122) were from the original EAPC recommendations and 13 were new statements including emerging specialisms: neonatal, geriatric and dementia care and better care practices. Seven statements failed to reach consensus after Round 3. Six related to specific occupational roles which were not regarded as essential to the multidisciplinary team; namely, occupational therapist, speech therapist, complementary therapist, lymphoedema therapist, trainer and librarian. Finally there was no consensus on population size that a volunteer team should serve. Four statements were removed as irrelevant or repetition; two after Round 1 (Supportive care as a synonym for palliative care and secondly an estimate of the ratio of cancer to non-cancer patients) and two after Round 2 (Hospital at home and a new item: non-specialist palliative care instead of the former term ‘general palliative care’). Of the 118 statements that reached consensus at Round 1, 107 were from the original standards and norms. 11 were new. Of the nine statements that reached consensus in Round 2, seven were revised statements from the original standard and norms. Two were new statements (Geriatric palliative care; dementia palliative care). All the statements that reached consensus in Round 3 were revised versions of the original standards and norms. The seven statements that did not reach consensus were original standards and norms. The following sections will briefly explain the overall results in each section.

**Table 2.** All consensus items from previous Norms and Standards.

Section	Question no	Item	Description	Round attained consensus	Results (%)			
					Agree	Neutral	Disagree	Don’t know
Section I: Terminology	1	Definition of Palliative Care	Palliative care is the active, total care of the patients whose disease is not responsive to curative treatment. Control of pain, of other symptoms, and of social, psychological and spiritual problems is paramount. Palliative care is interdisciplinary in its approach and encompasses the patient, the family and the community in its scope. In a sense, palliative care is to offer the most basic concept of care – that of providing for the needs of the patient wherever he or she is cared for, either at home or in the hospital. Palliative care affirms life and regards dying as a normal process; it neither hastens nor postpones death. It sets out to preserve the best possible quality of life until death (EAPC).	1	93	0	0	0
	2	Definition of Palliative Care Approach	Palliative care is an approach that improves the quality of life of patients and their families facing the problems associated with life-threatening illness, through the prevention and relief of suffering by means of early identification and impeccable assessment and treatment of pain and other problems, physical, psychosocial and spiritual (WHO).	1	86	5	9	0
	3	Definition of Hospice Care	Hospice Care is for the whole person, aiming to meet all needs - physical, emotional, social and spiritual. At home, in day care and in the hospice, they care for the person who is facing the end of life and for those who love them. Staff and volunteers work in multi-professional teams to provide care based on individual need and personal choice, striving to offer pain relief, dignity, peace and calm.	1	82	7	11	0
	4	Definition of Supportive care	Supportive care is the prevention and management of the adverse effects caused by treatment of life threatening and life limiting illnesses. This includes both physical and psychosocial symptoms, existential concerns and side effects across the entire continuum including the enhancement of rehabilitation and survivorship.	2	82	0	18	0
	6	Definition of End of Life Care	End-of-life care may be understood more specifically as comprehensive care for dying patients in the last few hours, days or weeks of life.	1	82	2	16	0
	7	Definition of Respite Care	Family members or other primary caregivers caring for a palliative care patient at home may suffer from the continuous burden of care. Respite care may offer these patients and their caregivers a planned or unplanned break.	1	93	5	0	2
Section II: The Philosophy of Palliative Care	8	Autonomy	In palliative care, the intrinsic value of each person as an autonomous and unique individual is acknowledged and respected. Care is only provided when the patient and/or family are prepared to accept it. Ideally, the patient preserves his/her self-determination regarding the power of decision making on place of care, treatment options and access to specialist palliative care.	1	98	0	2	0
	9	Dignity	Palliative care is supposed to be performed in a respectful, open and sensitive way, sensitive to personal, cultural and religious values, beliefs and practices as well as the law of each country.	1	95	0	5	0
	10	Relationship between patients and healthcare professionals	Palliative care staff should maintain a collaborative relationship with patients and families. Patients and families are important partners in planning their care and managing their illness.	1	98	0	2	0
	11	Quality of Life	A central goal of palliative care is to achieve, to support, to preserve and to enhance the best possible quality of life.	1	98	0	2	0
	12	Position towards life and death	Palliative care seeks neither to postpone nor hasten death.	1	92	4	4	0
	13	Communication	Good communication skills are an essential prerequisite for quality palliative care. Communication refers to the interaction between patient and healthcare professionals, but also to the interaction between patients and their relatives as well as the interaction between different healthcare professionals and services involved in the care.	1	100	0	0	0
	14	Public education	It is essential to build community capacity and to promote preventive healthcare that will leave future generations less afraid of the dying and bereavement that will confront all of us.	1	88	5	5	2
	15	Multiprofessional and interdisciplinary approach	Palliative care is supposed to be provided within a multiprofessional and interdisciplinary framework. Although the palliative care approach can be put into practice by a single person from a distinct profession or discipline, the complexity of specialist palliative care can only be met by continuous communication and collaboration between the different professions and disciplines in order to provide physical, psychological, social and spiritual support.	1	95	0	5	0
	16	Grief and bereavement	Grief and bereavement risk assessment is routine, developmentally appropriate and ongoing for the patient and family throughout the illness trajectory, recognising issues of loss and grief in living with a life-threatening illness. Bereavement services and follow-up support are made available to the family after the death of the patient.	1	95	0	5	0
Section III: Levels of Palliative Care	17	Levels of Palliative care	Palliative care can be delivered on different levels. At least two levels should be provided: a palliative care approach for non-specialised settings and specialist palliative care.	1	89	5	6	0
	18	Palliative Care Approach	The palliative care approach is a way to integrate palliative care methods and procedures in settings not specialist in palliative care This includes not only pharmacological and non-pharmacological measures for symptom control, but also communication with patient and family as well as with other healthcare professionals, decision making and goal setting in accordance with the principles of palliative care.	1	84	7	7	2
	20	Definition of General Palliative Care	The two-step ladder of care can be extended to three steps: a palliative care approach, generalist palliative care and specialist palliative care. A palliative care approach applies to those with limited experience and knowledge in dealing with palliative care but can apply the basic principles of good palliative care. Generalist palliative care applies to those who are frequently involved with palliative care and have some specialist palliative care knowledge, such as primary care clinicians, oncologists, and geriatricians, but do not provide palliative care as the sole purpose of their work. The third step would be specialist palliative care clinicians, whose sole role is to care for palliative patients.	2	87	5	8	0
	21	Specialist Palliative Care	Specialist palliative care is provided by specialised services for patients with complex problems not adequately covered by other treatment options. Specialist palliative care services require a team approach, combining a multi-professional team with an interdisciplinary mode of work. Team members must be highly qualified and should have their main focus of work in palliative care.	1	98	0	2	0
	24	Centres of Excellence	Centres of excellence in palliative care should act as a focus for education, research and dissemination, developing standards and new methods.	1	93	5	2	0
	25	Population served: Available to all with life-threatening diseases	Palliative care is not restricted to patients with predefined medical diagnoses but should be available for all patients with life-threatening diseases.	1	98	0	2	0
	26	Population served: Needs and Access	In all European countries, palliative care is predominately delivered to patients with advanced cancer. Patients with other diseases, such as neurological diseases, or with cardiac, pulmonary or renal failure, may have the same palliative care needs as cancer patients, but find it much harder to access palliative care	1	98	0	0	0
	27	Population served: National and European health policy priority	Providing access to high quality palliative care for non-cancer patients should be a priority of national and European health policy development.	1	95	5	2	0
	29	Disease stage: Appropriate for all with life threatening or life-limiting condition	Palliative care is appropriate for all patients from the time of diagnosis with a life-threatening or life limiting condition.	1	91	7	7	2
	30	Disease stage: Patients of all ages and diagnostic categories	The term life-threatening or life limiting illness here is assumed to encompass the population of patients of all ages and a broad range of diagnostic categories, who are living with a persistent or recurring condition that adversely affects daily functioning or will predictably reduce life expectancy.	1	86	5	5	0
	31	Children and adolescents: Closely related field to adult palliative care	Palliative care for children represents a special, albeit closely related field to adult palliative care (WHO).	1	93	2	5	5
	32	Children and adolescents: Begins at diagnosis with unit of care child and family	Palliative care for children begins when the illness is diagnosed and continues regardless of whether or not the child receives treatment directed at the disease. The unit of care is the child and family (EAPC).	1	88	2	0	2
	33	Children and adolescents: Specific paediatric palliative care services for inpatient and home treatment	Specific paediatric palliative care services for inpatient treatment and home care should be implemented. A full range of clinical and educational resources must be available for the child and family, in a format that is appropriate to age, cognitive and educational ability (EAPC).	1	96	2	2	2
	34	Children and adolescents: Family home and access to multi-disciplinary palliative care team	The family home should remain the centre of caring whenever possible. Every family should have access to multi-disciplinary, holistic paediatric palliative care team at home.	1	89	7	0	0
Section IV: Palliative Care Delivery	39	Advance Care Planning	Advance care planning enables patients to express their preferences and wishes for care in the context of conversations and written documents.	1	95	5	0	0
	42	Access to services: Availability of services	Services should be available to all patients, wherever and whenever they require them, without delay.	1	100	0	0	0
	43	Access to services: Equity	Equity of access to palliative care should be guaranteed in all European countries, assuring provision of palliative care according to needs and regardless of cultural, ethnic or other background.	1	100	0	0	0
	44	Access to services: High quality irrespective of ability to pay	Access to high-quality palliative care should not depend on the ability of patients or carers to pay.	1	100	0	0	0
	45	Preferred place of care	Patients may wish to be cared for in their own homes until the time of death, but some patients might prefer to remain in a hospice or other institution until death. It is important to elicit the preference of patients in regards to where they wish to receive care, and for this to be an on-going conversation between health professionals, patients and family members.	2	100	0	0	0
	46	Preferred place of care: Acknowledged and discussed with patient and family	The preferred place of care and place of death should be acknowledged and discussed with the patient and family, and measures taken to comply with these preferences, wherever possible.	1	98	2	0	0
	47	Preferred place of care: Treatment, care and support provided in all settings	Palliative treatment, care and support are provided at home, in nursing homes, in long term care facilities, in hospital and in hospice, or in other settings, if required.	1	95	5	5	5
	48	Non-specialist palliative care services. . . Community nursing services	NA	1	83	7	5	0
	49	Non-specialist palliative care services. . . General practitioners	NA	1	86	9	10	9
	50	Non-specialist palliative care services. . . Ambulant nursing services	NA	1	81	5	2	0
	51	Non-specialist palliative care services. . . General hospital units	NA	1	83	12	7	2
	52	Non-specialist palliative care services. . . Nursing homes	NA	1	77	14	2	0
	53	Specialist palliative care services. . . Inpatient palliative care unit	NA	1	98	0	2	5
	54	Specialist palliative care services. . . Inpatient hospices	NA	1	91	2	2	0
	55	Specialist palliative care services. . . Hospital palliative care support teams	NA	1	94	2	2	0
	56	Specialist palliative care services. . . Home palliative care teams	NA	1	98	0	2	7
	57	Specialist palliative care services. . . Community hospice teams	NA	1	82	9	2	5
	58	Specialist palliative care services. . . Day hospices	NA	1	83	10	8	3
	60	Specialist palliative care services. . . Palliative care clinics	Defined as outpatient clinics dedicated to the management of palliative care patients	2	81	8	5	0
	61	Access to specialist advice and support by non-specialist services	Now, as in the future, a major part of palliative care will be provided by non-specialist services. Consequently, non-specialist professionals must have easy access to specialist consultation for advice and support.	1	95	0	7	2
	62	Fast-tracking care pathways	Considering the reduced life expectancy of palliative care patients, fast-tracking care pathways should be implemented in medical services, ensuring adequate priority for these patients, to prevent disproportional burden from lost time.	1	86	5	2	0
	63	Integrated system of services	A comprehensive integrated system of services, including inpatient services, home-care services and support services, should be available to cover all care needs and treatment options.	1	95	2	0	0
	64	Graded system of palliative care services	In a graded system of palliative care services, the different needs of the patients and their caregivers can be matched with the most suitable service. With such a system, the right patients can be treated at the right time in the right place.	1	91	9	0	0
	65	Palliative Care Networks: Integrating a broad spectrum of institutions and services	Regional networks integrating a broad spectrum of institutions and services, and effective coordination, will improve access to palliative care and increase quality as well as continuity of care.	1	98	2	0	5
	66	Palliative Care Networks: At least one specialist palliative care inpatient unit within each healthcare service area	There should be at least one specialist palliative care inpatient unit within each healthcare service area.	1	88	7	7	2
	67	Palliative Care Networks: Specialist palliative care inpatient unit	The specialist palliative care inpatient unit should be amongst the core elements of the specialist palliative care service. Other community settings and locations are also important elements of the specialist palliative care service.	3	87	5	8	0
	68	Palliative Care Networks: Effective coordination	Effective coordination is best achieved with a case manager (case coordinator, key worker) who can provide transfer of information and continuity of care across different settings. An effective coordination of services will allow a higher proportion of people to die at home if they so wish.	1	77	14	0	5
	69	Palliative Care Networks: Availability of coordination services	To be effective, these coordination services must be available 24 hours a day, seven days a week.	1	86	9	0	5
	70	Palliative Care Networks: Coordination management	Coordination can be accomplished by a team or a person. Case management and coordination can be performed by an interdisciplinary expert group representing the various services within the network, or by a palliative care unit or an inpatient hospice.	1	88	7	5	5
	71	Palliative Care Network tasks. . .	Consensual definitions of goals and quality standards	1	85	5	12	5
	72	Palliative Care Network tasks. . .	Uniform criteria for admission and discharge at all levels of care	1	78	5	5	0
	73	Palliative Care Network tasks. . .	Use of common evaluation methods	1	83	12	9	2
	74	Palliative Care Network tasks. . .	Implementation of common therapeutic strategies based on available evidence	1	82	7	7	0
	75	Staff in specialist palliative care services: support to implement a palliative care approach	Services that are not specialised in palliative care can use a palliative care approach or deliver general palliative care, even when done by one professional category, or even by one individual (for example, a general practitioner working alone), if they have access to support from an interdisciplinary team.	1	86	7	0	0
	76	Staff in specialist palliative care services: Specialist palliative care service requires multiprofessional and interdisciplinary work style	Specialist palliative care service delivery requires a multiprofessional team with an interdisciplinary work style.	1	100	0	0	0
	77	Necessity of professional groups for specialist palliative care services. . . Physiotherapist	NA	1	98	2	0	2
	79	Necessity of professional groups for specialist palliative care services. . . Social Worker	NA	1	98	0	0	0
	80	Necessity of professional groups for specialist palliative care services. . . Professionals skilled in psychosocial support	NA	1	100	0	2	0
	81	Necessity of professional groups for specialist palliative care services. . . Professionals skilled in bereavement support	NA	1	94	2	5	0
	82	Necessity of professional groups for specialist palliative care services. . . Coordinator for spiritual care	NA	1	79	16	2	0
	84	Necessity of professional groups for specialist palliative care services. . . Dietician	NA	1	79	19	2	2
	85	Necessity of professional groups for specialist palliative care services. . . Pharmacist	NA	1	87	9	0	0
	87	Necessity of professional groups for specialist palliative care services. . . Coordinator for voluntary workers	NA	1	79	21	0	0
	89	Necessity of professional groups for specialist palliative care service. . . Wound Management Specialist	NA	3	76	18	6	0
	91	Necessity of professional groups for specialist palliative care services. . . Chaplain	NA	1	91	9	2	0
	93	Necessity of professional groups for specialist palliative care services. . . Adequate number of office workers, administration secretaries and general assistants	NA	1	93	5	10	0
	94	Staff in specialist palliative care services: Core palliative care team	The core palliative care team should consist of nurses and physicians with special training as a minimum, supplemented by psychologists, social worker and physiotherapists if possible. Other professionals can be members of the core team, but more frequently will work in liaison.	1	88	2	0	7
	95	Staff in specialist palliative care services: Paediatric palliative care services	Children need specialised services which are to be provided by paediatrically skilled staff. This applies especially for the paediatric palliative care nursing service. Palliative home care for the support of children and their families should be available.	1	91	2	0	0
	96	Staff in specialist palliative care services: Role of volunteers	Specialist palliative care services should include volunteers or collaborate with volunteer services.	1	93	7	5	0
Section V: Palliative Care Services	97	Definition and purpose of palliative care units	Palliative care units (PCU) provide specialist inpatient care. A palliative care unit is a department specialised in the treatment and care of palliative care patients. It usually is a ward within or adjacent to a hospital but can also exist as a stand-alone service.	1	93	2	7	5
	98	Definition and purpose of palliative care units II	In some countries palliative care units will be regular units in hospitals, providing crisis intervention for patients with complex symptoms and problems, in other countries PCU can also be freestanding institutions, providing end-of-life care for patients where home care is no longer possible.	1	76	12	5	0
	99	Definition and purpose of palliative care units III	The aim of palliative care units is the alleviation of disease- and therapy-related discomfort and, if possible, to stabilize the functional status of the patient and offer patients and carers psychological and social support in a way that allows for discharge or transfer to another care setting.	1	90	5	5	2
	100	Demand of Palliative Care Units	An aspirational norm and standard is that there should be 80-100 palliative care and hospice beds per 1.000.000 inhabitants. Using a 8-12 bed PCU as an example, this would correspond to 8-10 PCUs per million inhabitants. However, this also must consider local demand and institutional structure.	3	86	3	8	3
	101	Requirements of palliative care units	PCUs require a highly qualified, multidisciplinary team. Staff members in palliative care units are supposed to have specialist training. The core team consists of physicians and nurses. The extended team comprises relevant associated professionals, such as psychologists, physiotherapists, social workers or chaplains.	1	93	0	7	0
	102	Requirements of palliative care units II	There should be regular multidisciplinary team meetings (minimum weekly) to review palliative care patient’s referrals and care plans.	1	93	0	3	8
	103	Palliative Care Unit Requirements III	PCUs require a dedicated core team of nurses and physicians. Qualified nursing staff should encompass a shift ratio of at least one nurse per bed, and preferably 1.2 nurses per bed. PCUs require physicians and nurses with special training, with 1 physicians per 5-7 beds. In a setting where children are being cared for, there should be at least one nurse on each shift with a special paediatric qualification.	2	81	8	7	0
	104	Requirements of palliative care units III	Palliative care units should offer a homelike atmosphere with quiet and private areas. They should be separate areas with a capacity of 8–15 beds. The units should be equipped with single or double patient rooms, facilities for relatives to stay overnight and rooms for social activities, such as kitchens or living rooms.	1	93	2	10	5
	105	Definition and purpose of inpatient hospice	An inpatient hospice admits patients with palliative care and end of life care needs when treatment in a hospital is not necessary or desired, and care at home or in a nursing home is not possible.	1	77	8	3	3
	106	Inpatient Hospice Requirements	An inpatient hospice requires a multiprofessional team that cares for patients and their relatives using a holistic approach. Nursing staff should encompass at least one, preferably 1.2 nurses per bed. A physician specialised in palliative care should be available 24 hours a day and be accessible within 30 minutes. There should be dedicated input from healthcare professionals that can provide support for the patient’s physical, psychosocial and spiritual needs. This should be supported by a team of voluntary workers.	2	81	13	8	5
	107	Requirements of inpatient hospice II	An inpatient hospice requires a homelike atmosphere with access for people with disabilities, single or double patient rooms and a capacity of at least eight beds. The hospice should be equipped with rooms for social and therapeutic activities.	1	82	5	0	0
	108	Definition and purpose of hospital palliative care support team	Hospital palliative care support teams provide specialist palliative care advice and support to other clinical staff, patients and their families and carers in the hospital environment. They offer formal and informal education and liaise with other services in and out of the hospital.	1	98	2	0	0
	109	Definition and purpose of hospital palliative care support team II	One central aim of a hospital palliative care support team is the assessment of palliative care needs and alleviation of symptoms of patients on different hospital wards. Also, the team offers mentoring of the attending staff and support to patients and their relatives. Furthermore, expertise in palliative medicine and palliative care shall be made available in the respective environments.	1	97	3	3	3
	110	Definition and purpose of hospital palliative care support team III	The aims of a hospital palliative care support team are the improvement of palliative care and to foster discharge from an acute hospital unit and the facilitation of the transfer between inpatient and outpatient care.	1	91	3	0	0
	111	Demand of hospital palliative care support team	A hospital palliative care support team should be affiliated to every PCU and should be available for every general hospital in case of need.	1	92	8	2	5
	112	Demand of hospital palliative care support team II	There should be at least one team available for a hospital with 250 beds.	1	86	7	5	0
	113	Requirements of hospital palliative care support team	A hospital palliative care support team is composed of a multiprofessional team with at least one nurse and one physician with specialist palliative care training.	1	92	3	2	0
	114	Requirements of hospital palliative care support team II	A hospital palliative care support team should have a room for staff meetings and administrative support at its disposal.	1	95	3	0	2
	115	Definition and purpose of home palliative care team	Home palliative care teams provide specialised palliative care to patients who need it at home and support to their families and carers at the patient’s home. They also provide specialist advice to general practitioners, family physicians and nurses caring for the patient at home.	1	95	3	3	3
	116	Definition and purpose of home palliative care team II	The home palliative care team also assists the transfer between hospital and home care.	1	91	3	0	11
	117	Demand of home palliative care team	There should be one home palliative care team available for 100,000 inhabitants. The team should be accessible 24 hours a day.	1	84	5	10	2
	118	Requirements of home palliative care team	The core team of a Home Palliative Care Team consists of 4 – 5 whole time equivalent professionals and comprises nurses and physicians with specialist training, a social worker and administrative staff.	1	78	10	0	0
	119	Requirements of home palliative care team II	The home palliative care team works in close collaboration with other professionals so that the full range of multiprofessional teamwork can be realised in the home-care setting.	1	95	5	3	0
	120	Requirements of home palliative care team III	Palliative care at home requires close collaboration of other professional services, such as specialised nursing services and general practitioners with specialist training, including (but not restricted to) regular meetings at the patient’s bedside.	1	95	2	3	0
	121	Requirements of home palliative care team IV	The home palliative care team requires a working room at its disposition for nurses, physicians and social workers, as well as a meeting room and a depot for medical equipment and supplies.	1	92	5	0	15
	122	Definition and purpose of Hospital at home (Hospice@Home)	The hospital at home provides intensive palliative care for the patient at home.	1	75	10	10	2
	123	Definition and purpose of volunteer hospice team	A volunteer hospice team offers support and befriending to palliative care patients and their families in times of disease, pain, grief and bereavement.	1	80	8	0	10
	124	Definition and purpose of volunteer hospice team II	The volunteer hospice team is part of a comprehensive support network and collaborates closely with other professional services in palliative care.	1	80	10	2	10
	126	Requirements of volunteer hospice team	The volunteer hospice team comprises specially trained voluntary hospice workers with at least one professional co-ordinator.	1	85	3	5	8
	127	Requirements of Volunteer Hospice Team II	Rates of volunteering vary significantly across Europe due to cultural, financial, and institutional reasons. As a result, we propose that a volunteer hospice team providing direct support should consist of at least 10 to 12 volunteer hospice workers and one dedicated professional (or volunteer) co-ordinator.	3	86	8	0	6
	128	Requirements of volunteer hospice team IV	The co-ordinator should have expertise in the social- and/or health sector with additional specialist training in palliative care.	1	82	5	2	5
	129	Requirements of volunteer hospice team III	The voluntary workers should have participated in an accredited instruction course and take part in regular supervision and self-reflection, as well as continuing education.	1	88	5	3	10
	130	Definition and purpose of Day hospice	Day hospice or day care centre are spaces in hospitals, hospices, palliative care units or the community especially designed to promote recreational and therapeutic activities among palliative care patients.	1	85	2	0	8
	131	Definition and purpose of Day hospice II	Central aims are social interaction and therapeutic care, to avoid social isolation as well as to relieve the burden of care on relatives and caregivers.	1	85	7	0	7
	132	Demand of Day Hospice	A day hospice is defined as a palliative care centre providing specific medical management/treatment. There should be a day hospice available for 100,000 - 150,000 inhabitants.	3	81	8	3	8
	133	Requirements of Day care centre	A day care centre is staffed by a multiprofessional team supplemented by voluntary workers.	1	88	5	2	5
	134	Requirements of Day Care Centre II	A day care centre is defined as a centre specialising in more holistic and psychosocial palliative care. It is recommended that there are two health or social care workers present during opening hours, with at least one being a specialist palliative care professional who capable of assessing patients and seeking further medical assistance if required.	3	86	8	3	3
	135	Requirements of Day care centre III	A qualified physician should be directly accessible in case of need. Ready access to other professionals, such as physiotherapists, social workers or spiritual care workers, should be obtained.	1	90	3	2.5	7.5
	136	Requirements of Day care centre IV	A day care centre is supposed to have patient rooms, a therapy room, staff rooms, a bathroom, a kitchen and a recreation room. All rooms should have access for people with disabilities.	1	82.5	7.5	0	10
	137	Requirements of Day Care Centre V	A day care centre is defined as a centre specialising in more holistic and psychosocial palliative care. A day care centre is a unit with a minimum of 4 places. A day care centre may be associated or located within an inpatient hospice, palliative care unit or other care facility.	3	84	8	3	5
	138	Definition and purpose of palliative outpatient clinic	Palliative outpatient clinics are an important component of a community palliative care programme.	1	83	7	5.5	5.5
	139	Definition and purpose of palliative outpatient clinics II	Usually outpatient clinics are affiliated to specialist PCUs (in hospitals), inpatient hospices or specific community services. This aims to provide continuity and support to those whose performance status is deteriorating.	2	76	13	2	13
	140	Definition and purpose of palliative outpatient clinic III	Palliative outpatient clinics should be integrated in regional networks, in order to consult with inpatient services, home palliative care team or the primary care team.	1	78	8	2	12

**Table 3.** Thirteen new Standard and Norm statements not in original 2009 recommendations.

Section	Question no	Statement	Description	Round attained consensus	Results			
					Agree	Neutral	Disagree	Don’t know
Section III: Levels of Palliative Care	22	Definition of Geriatric Palliative Care	Geriatric Palliative Care (GPC) has recently been identified and defined as ‘. . .a field of interspecialty collaboration unifying competences from geriatric medicine and palliative care to respond to the socio-demographic changes and challenges of older adults with severe and life-limiting conditions’. * Geriatric palliative care should be part of the responsibility of both palliative and geriatric care specialties. * Voumard, R., Rubli Truchard, E., Benaroyo, L. et al. Geriatric palliative care: a view of its concept, challenges and strategies. BMC Geriatr 18, 220 (2018). https://doi.org/10.1186/s12877-018-0914-0	2	89	0	11	0
	23	Dementia Palliative Care	Caring for a person at the end of life who has dementia can be complex and patients and families may need palliative care specific to dementia. The EAPC have attained consensus on eleven important components needed for optimal palliative care in older people with Dementia*. Caring for palliative dementia patients can be challenging, and sometimes specialist dementia palliative care input is required. * van der Steen JT, Radbruch L, Hertogh CM, et al. White paper defining optimal palliative care in older people with dementia: a Delphi study and recommendations from the European Association for Palliative Care. Palliat Med. 2014;28(3):197-209. doi:10.1177/0269216313493685	2	87	0	8	5
	35	Children and adolescents: Palliative Care for neonates a closely related field	Palliative care for neonates represents a special, albeit closely related field to paediatric palliative care.	1	86	9	0	5
	36	Perinatal Palliative Care	General care designed not only to minimise pain in neonates but also to make them more comfortable, promote individualised developmental care [6] and facilitate bonding with the mother are critical components of perinatal palliative care	1	79	9	0	12
	37	Early initiation of perinatal palliative care	Early initiation, starting from diagnosis, of perinatal palliative care is important to parents who must cope with a tragic prenatal diagnosis.	1	81	7	0	12
	38	Perinatal Palliative Care can be provided in existing settings	Services can be provided in existing settings, including specialist settings, and should focus on the needs of the foetus, mother and the psychological, spiritual and social needs of the whole family.	1	79	9	0	12
Section IV: Palliative Care Delivery	40	Access to services: Information directory	People should have access to a national directory of information on palliative care providers, local caregivers and other relevant organisations that can have a role in palliative care.	1	98	2	0	0
	41	Access to services: Websites	All palliative care services should have a website	1	91	7	2	0
Section V: Palliative Care Services	141	Guidelines in all settings: opening hours	There should be unrestricted open hours for the family and friends of dying patients.	1	100	0	0	0
	142	Guidelines in all settings: access to opioids and other essential medicines	There should be access to opioids and other essential medicines routinely used in palliative care in all settings.	1	97	3	0	0
	143	Guidelines in all settings: information exchange across caregivers, disciplines and settings	There should be a process to support the exchange of information across caregivers, disciplines and settings.	1	95	5	0	0
	144	Guidelines in all settings: digital medical records	There should be a digital medical record, to which all professional caregivers involved in the care of palliative care patients have access within one setting.	1	97	3	0	0
	145	Guidelines in all settings: availability of specialist equipment in all settings	Specialist equipment (e.g. anti-decubitus mattresses, aspiration material, stoma care, oxygen delivery, special drug hospital beds) should be available for the care of palliative care patients in each specific setting.	1	100	0	0	0

**Table 4.** All statements that failed to reach consensus.

Section	Question no	Statement description	Description	Round 3 Result			
				Agree	Neutral	Disagree	Don’t know
Section IV: Palliative Care Delivery	78	Necessity of professional groups for specialist palliative care services. . . Occupational Therapist	NA	61	21	8	0
	83	Necessity of professional groups for specialist palliative care service. . . Speech Therapist	NA	73	8	19	0
	86	Necessity of professional groups for specialist palliative care services. . . Complementary Therapist	NA	55	26	16	3
	88	Necessity of professional groups for specialist palliative care services. . . Trainer/Instructor	NA	39	26	29	5
	90	Necessity of professional groups for specialist palliative care services. . . Lyphoedema Specialist	NA	73	11	13	3
	92	Necessity of professional groups for specialist palliative care services. . . Librarian	NA	27	21	52	0
Section V: Palliative Care Services	125	Demand of volunteer hospice team	As an aspirational norm and standard across Europe, there should be one volunteer hospice team available for 30,000 - 40,000 inhabitants (or a proportional equivalent - e.g. a larger team for a larger population).	72	11	6	11

#### Definition and terminology of palliative care and hospice care

Overall six of the seven original statements relating to terminology in palliative care continued to be strongly endorsed in Round 1, especially the previous EAPC definition with 93% agreement compared to the WHO definition with 86% agreement. The term ‘non-specialist palliative care’ instead of ‘general’ palliative care had been identified in the literature review^15^ but qualitative comments indicated it was confusing.

#### Philosophy of palliative care

Nine original statements about the common principles of palliative care reached very high consensus (greater than 90%) in Round 1 such as the values of patient autonomy and dignity. Only the statement on the need for public education (88%) was slightly lower.

#### Levels of palliative care

Twenty (6 new, 14 original) of the 22 statements on the nature and scope of palliative care reached consensus, 17 in Round 1 and a further 3 in Round 2. There was a high level (over 90%) of agreement on six statements such as the nature of specialist palliative care, need for centres of excellence and that palliative care should be available for all diagnoses. There was consensus on four new statements that recognised the emerging role of neonatal palliative care. Two statements were withdrawn, one on the need for services to include non-cancer patients which repeated another statement, and a new term, ‘non-specialist’ palliative care.

#### Palliative care delivery

This is the largest section with 47 (2 new, 45 original) statements reaching consensus in Round 1, with two further statements gaining consensus in both subsequent rounds. There was high levels (over 90%) agreement for 24 statements including the importance of advance care planning, equity in access to services, facilitating preferred place of care and service provision including specialist palliative care, hospital teams, in-patient units and home care. Two new statements relating to access to services, the availability of national directories and websites, indicates that information provision is crucial. Six statements that failed to reach consensus were related to specific occupational roles which were not regarded as essential in the multidisciplinary team; occupational therapist, speech therapist, complementary therapist, lyphoedema therapist, trainer and librarian. One statement ‘hospital at home’ was withdrawn at round 2 as it was regarded as ambiguous.

#### Palliative care services

This section concerned specific types of palliative care services with 40 (5 new, 35 original) statements reaching consensus in Round 1, followed by three in Round 2 and five in Round 3. There were high levels (over 90%) of agreement for 21 statements such as the purpose and staffing requirements of specialist palliative care units, hospital palliative care teams and home palliative care teams. There were lower levels of agreement on the purpose and requirements for Day Care services, reflecting differences within Europe. There was a high level of agreement on five new statements which offered guidelines on open visiting, availability of essential medicines, better information exchange, including digital medical records and access to specialist equipment. One statement on the configuration of volunteer teams failed to reach consensus.

*Expert consultation*: The EAPC Board approved the updated recommendations, and some provided editorial comments.

## Discussion

### Summarising findings

This revision to the EAPC recommendations on standards and norms in palliative care identified that there remains a high consensus amongst European experts with the original recommendations.^8,9^ The core principles of palliative care are fully endorsed, along with shared understanding about definitions of palliative care and how these are articulated in specialist palliative care services. It has highlighted the emergence of new areas of specialisation in palliative care including neonatal palliative care,^17^ geriatric and dementia services.^18,19^ These attest to the needs of patients across the life span as recommended by WHO.^5^ Also there is greater awareness of the need to improve access to better information transfer and the role of digital health technologies including electronic health records.^20^

In comparison with the previous study,^8,9^ there were responses from organisations in just over half of all countries in Europe 27/52 – 52%, rather than 21 countries, reflecting increases in EAPC membership 52 compared to 35.^21^ There were more female respondents, 68% compared to 48% indicating both a shift in medicine and in healthcare. As before, the majority of respondents were physicians or other clinicians. There remains strong endorsement of definitions for palliative care derived from the EAPC and WHO, despite alternative definitions being proposed in the interim such as severe health-related suffering.^10,11^ These may be more appropriate in low and middle income countries where palliative care is less well developed than in Europe. The term ‘supportive care’ was not regarded as a synonym for palliative care, although this is advocated in the USA.^2^ As palliative care develops, it is perhaps appropriate that the diversity of geopolitical, health system, population needs and economic resources in specific regions and countries is recognised; we do not seek to offer a global definition or standards, merely to facilitate collaboration in Europe.

European countries are at remarkably different stages in their development of palliative care as demonstrated in the EAPC Atlas.^12^ It is thus rather challenging to provide a balance of standards and norms that accommodates such diversity. Especially in eastern Europe, there are countries which are at very early stages in pioneering palliative care services, while typically in western countries, services are already well established and integrated in national healthcare systems. Here concerns about ensuring equity and access for marginalised populations have become one of the predominant issues. However, the major drivers to address WHO directives to strengthen palliative care, include it as part of universal health coverage and embed services in national healthcare systems appear to still focus on extending palliative care beyond cancer.^5^ Recent guidance from the WHO on how to establish the foundations and structure of palliative care services are likely to improve quality but need to be adopted, resourced and implemented within countries,^22^ and progress towards these goals should be assessed in further research.

The specific values on the philosophy of palliative care demonstrate a strong consensus across European experts, with a recognition that palliative care is suitable for all patients regardless of diagnosis. However this may belie continuing evidence of inequalities in access to hospice care.^23^ The survey shows that there remains some geographical diversity in how services are configured, probably reflecting their differing historical origins, especially with volunteers.^24^ Volunteer roles differ across Europe such as to provided direct hands on care, to supplement services, or in a few countries to fund-raise for hospices,^24^ and these differences might explain why consensus was not attained. The survey confirms that there is still a lack of consensus on whether some staff roles are essential or merely desirable additions to the multidisciplinary team, specifically certain therapists and educationalists. This probably reflects diversity in development and whether education are considered to be supplementary activities.

Our respondents demonstrated high consensus on statements relating to specialist palliative care in hospital and home care settings rather than ‘general palliative care’ provided by non-specialists. A recently proposed term ‘non-specialist palliative care’ instead of general palliative care was not favoured,^25^ and reflects the difficulty in making sharp distinctions between specialist and general provision.^21^ In some countries palliative care has emerged as a specific medical speciality, while in others it forms part of the work of medical professionals who are specialists in oncology or other areas.^26^

### Strengths and limitations

This study’s strengths include adherence to Delphi technique guidance^13^ and was conducted by a multidisciplinary team. Whilst a scoping of the literature was undertaken, a full systematic review might have identified additional concepts and practices, and this methodology should be considered for future revisions of the recommendations on standards and norms. We built upon the 2009 recommendations with a sample drawn from 30 organisations across Europe. There was a reasonable response rate of 58% organisations, and 100% in the expert consultation. As the survey was only available in English, this may have resulted in bias. Incentivisation (also previously used^8,9^), may have influenced the completion rate. Further research is required to elicit service-user views. Attrition over the Delphi rounds was low, but the impact of the Covid-19 pandemic on the initial response was probably greater for those with clinical responsibilities during the winter of 2020/2021.^27^

### What this study adds

There is evidence of emerging specialisation of palliative care services in neonatal, geriatrics and dementia. A balance is needed between tailoring services to meet local population needs and the integration of services within healthcare systems.^6^ Specialist palliative care providers need to demonstrate flexibility, as during the pandemic.^28^ As before, the recommendations provide evidence for benchmaking palliative care services and promoting consistency in Europe. The standards and norms offer opportunities to enhance advocacy and collaborative health policies, to promote better use of resources and offer equitable provision of palliative care for patients and families. Our findings have implications for many stakeholders including designing palliative care service improvements, advocating for better integration of palliative care and ensuring that palliative care is included in new strategies and policies.

## Conclusions

Most recommendations on standards and norms for palliative care in Europe remain unchanged since 2009, with 13 new recommendations relating to emerging specialisms and service improvements. Evolving concepts can be used to support advocacy. Further research is needed to understand how these standards and norms are utilised to deliver quality improvements.

## Supplemental Material

sj-pdf-1-pmj-10.1177_02692163221074547 – Supplemental material for Revised recommendations on standards and norms for palliative care in Europe from the European Association for Palliative Care (EAPC): A Delphi study Click here for additional data file. Supplemental material, sj-pdf-1-pmj-10.1177_02692163221074547 for Revised recommendations on standards and norms for palliative care in Europe from the European Association for Palliative Care (EAPC): A Delphi study by Sheila Payne, Andrew Harding, Tom Williams, Julie Ling and Christoph Ostgathe in Palliative Medicine
