# Supplementary material for: Revised recommendations on standards and norms for palliative care in Europe from the European Association for Palliative Care (EAPC): A Delphi study
Source: Palliat Med. 2022 Feb 3;36(4):680–97. doi: 10.1177/02692163221074547 (PMC9006395; doi:10.1177/02692163221074547)
Supplement: sj-pdf-1-pmj-10.1177_02692163221074547 – Supplemental material for Revised recommendations on standards and norms for palliative care in Europe from the European Association for Palliative Care (EAPC): A Delphi study [file sj-pdf-1-pmj-10.1177_02692163221074547.pdf]

Supplementary file 1: List of selected papers identified in scoping search

Balaguer A, Martín-Ancel A, Ortigoza-Escobar D, et al. The model of palliative care in the perinatal setting: a review of the literature. BMC Pediatr 2012; **12,** 25: doi.org/10.1186/1471-2431-12-25

Barazzetti G, Borreani C, Miccinesi G, et al. What "best practice" could be in Palliative Care: an analysis of statements on practice and ethics expressed by the main Health Organizations. BMC Palliat Care 2010; 9, 1: doi.org/10.1186/1472-684X-9-1

Callaway MV, Connor SR, Foley KM. World Health Organization Public Health Model: A Roadmap for Palliative Care Development. J Pain Symptom Manage 2018;55,2S:S6-S13: doi:10.1016/j.jpainsymman.2017.03.030

Claessen SJJ, Francke AL, Belarbi, HE et al. A new set of quality indicators for palliative care: process and results of the development trajectory. J Pain Symptom Manage 2011; 42, 2: 169-182.

De Roo ML, Leemans K, Claessen SJ, et al. Quality indicators for palliative care: update of a systematic review. J Pain Symptom Manage 2013. 46,4:556-72. doi: 10.1016/j.jpainsymman.2012.09.013.

Gómez-Batiste X, Stjernsward J, Espinosa J, et al. How to design and implement palliative care public health programmes: foundation measures. An operational paper by the WHO Collaborating Centre for Public Health Palliative Care Programmes at the Catalan Institute of Oncology. BMJ Supportive & Palliative Care 2013;**3:**18-25.

Hui D, Nooruddin Z, Didwaniya N, et al. Concepts and definitions for "actively dying," "end of life," "terminally ill," "terminal care," and "transition of care": a systematic review. J Pain Symptom Manage 2014; 47, 1: 77-89

Iliffe S, Davies N, Manthorpe J, et al. Improving palliative care in selected settings in England using quality indicators: a realist evaluation. BMC Palliat Care 2016;15:69: doi:10.1186/s12904-016-0144-1

Leemans K, Deliens L, Van den Block L, et al. Systematic Quality Monitoring For Specialized Palliative Care Services: Development of a Minimal Set of Quality Indicators for Palliative Care Study (QPAC). Am J Hosp Palliat Care 2017;34, 6:532-546. doi: 10.1177/1049909116642174.

Nevin M, Smith V, Hynes G. Non-specialist palliative care: A principle-based concept analysis. Palliative Medicine 2019;33, 6 :634-649. doi:10.1177/0269216319840963

Pfaff K, Markaki A. Compassionate collaborative care: an integrative review of quality indicators in end-of-life care. BMC Palliat Care 2017; 16, 65: 10.1186/s12904-017-0246-4

Radbruch L, Leget C, Bahr P, et al. Euthanasia and physician-assisted suicide: A white paper from the European Association for Palliative Care. Palliative Medicine 2015; 30, 2: 106-116.

van der Steen JT, Radbruch L, Hertogh CM, et al. White paper defining optimal palliative care in older people with dementia: A Delphi study and recommendations from the European Association for Palliative Care. Palliative Medicine 2014;28,3:197-209. doi:10.1177/0269216313493685

Voumard R, Rubli Truchard E, Benaroyo L, Borasio GD, Büla C, Jox RJ. Geriatric palliative care: a view of its concept, challenges and strategies. BMC Geriatr 2018;18, 1: 220. doi:10.1186/s12877-018-0914-0

Woitha K, Van Beek K, Ahmed N, et al. Development of a set of process and structure indicators for palliative care: the Europall project. BMC Health Services Research 2012; 12, 381: 10.1186/1472-6963-12-381

Woitha K, Van Beek K, Ahmed N, et al. Validation of quality indicators for the organization of palliative care: A modified RAND Delphi study in seven European countries (the Europall project). Palliative Medicine 2014; 28, 2:121-129. doi:10.1177/0269216313493952
